# Oral microbiome diversity and all-cause mortality in hypertensive adults: findings from a nationally representative cohort

**DOI:** 10.1080/20002297.2025.2609456

**Published:** 2026-01-02

**Authors:** Zhe Zhou, Zichao Zhuang, Yipeng Ding, Yufan Jiang, Shu Chen, Qinglian Zhang, Hanxin Que, Jian Lin, Hui Deng, Yi Wang

**Affiliations:** a Institute of Stomatology, School and Hospital of Stomatology, Wenzhou Medical University, Wenzhou, China; b Department of Periodontology, School and Hospital of Stomatology, Wenzhou Medical University, Wenzhou, China; c Department of Orthodontics, School and Hospital of Stomatology, Wenzhou Medical University, Wenzhou, China

**Keywords:** Oral microbiome diversity, hypertension, all-cause mortality, NHANES, survival analysis

## Abstract

**Background:**

Oral microbiome diversity has been associated with general health. However, its association with long-term outcomes in hypertensive individuals remains unclear.

**Objectives:**

This study aimed to investigate whether oral microbiome diversity is associated with all-cause mortality in hypertensive individuals.

**Design:**

Data from 2,669 hypertensive individuals in the National Health and Nutrition Examination Survey (NHANES, 2009–2012) were analyzed. Oral microbiome diversity was assessed using four alpha-diversity metrics: the Simpson index, Shannon–Weiner index, Faith’s Phylogenetic Diversity, and observed amplicon sequence variants (ASVs). Weighted multivariable Cox proportional hazards regression and interaction analyses were conducted.

**Results:**

During a mean follow-up of 8.61 years, 268 all-cause deaths occurred. Higher oral microbiome diversity assessed by the Simpson index (hazard ratio [HR] = 0.38; 95% confidence interval [CI], 0.20–0.75; *P*_trend_ < 0.01) and Shannon–Weiner index (HR = 0.47; 95% CI, 0.25–0.88; *P*_trend_ < 0.05), was significantly associated with reduction in all-cause mortality risk. A potential interaction between sex and oral microbiome diversity on mortality risk was observed.

**Conclusions:**

Higher oral microbiome diversity is an independent protective factor for survival in patients with hypertension, with potential sex-specific differences in this association. These findings suggest that enhancing oral microbiome diversity may potentially help promote overall health in individuals with hypertension.

## Introduction

The human microbiome, an intricate ecosystem harbouring trillions of microorganisms, plays a vital role in sustaining host homoeostasis through metabolic cross-talk and immunomodulation. The gut microbiome, in particular, has been demonstrated to account for systemic health outcomes such as cancer, cardiovascular diseases (CVDs) and metabolic syndromes [1–3]. This capacity has positioned faecal microbial diversity as both a diagnostic biomarker and potential therapeutic target in precision medicine approaches for chronic diseases. In comparison, the oral microbiome, being the second most diverse microbial niche in the body [4], remains relatively underexplored in the contexts of systemic disease. In addition to its well-established contributions to local conditions like periodontitis and caries [5,6], recent studies have shown a close relationship between oral microbiome and extraoral disorders including CVDs, diabetes, rheumatoid arthritis, Alzheimer’s disease and cancer [7–11]. However, evidence elucidating the role of microbiome in shaping the long-term clinical outcomes of chronic diseases remains limited.

Oral microbiome diversity, particularly alpha diversity which represents microbial richness and evenness within an individual, is recognised as an important characteristic of a healthy microbiome [12]. A reduction in the diversity of the oral microbiome may facilitate hematogenous dissemination of pathogenic oral microbiota, thereby driving persistent systemic low-grade inflammation [13]. Emerging evidence suggests that variations in oral microbiome diversity correlate with the onset and progression of multiple systemic disorders. Prior study has shown that aged individuals with compromised health status exhibit reduced salivary microbiome *α*-diversity [14]. Furthermore, a diminished oral microbiome *α*-diversity has been found in individuals with hyperthyroidism [15], lung cancer [16], and diabetes mellitus [17]. Nevertheless, most of these studies were cross-sectional or case-control with limited sample size. Critical gaps persist in characterising the temporal associations between oral microbiome diversity and clinical endpoints.

Recent National Health and Nutrition Examination Survey (NHANES) with longitudinal data have provided a valuable opportunity to investigate the long-term health effects of the oral microbiome at a population level. Previous studies have mainly examined the relationship between oral microbiome diversity and mortality in the general population [18,19], but evidence specific to hypertensive individuals remains scarce. Therefore, our study focused on adults with hypertension because this group represents a high-risk population with elevated mortality [20]. Biologically, individuals with hypertension are prone to endothelial dysfunction and systemic low-grade inflammation [21,22]. Oral microbiome dysbiosis has also been shown to contribute to systemic inflammation, endothelial dysfunction, immune dysregulation, and metabolic disturbances [23,24], partly mediated by translocation of microbial products and activation of host inflammatory pathways [25]. These mechanisms parallel the pathophysiological changes observed in hypertension, suggesting that the two conditions may act synergistically to exacerbate systemic disturbances and ultimately increase mortality risk. Motivated by these considerations, we tested the hypothesis that lower oral microbiome diversity is associated with higher mortality among hypertensive adults.

Building on these biological and epidemiological considerations, the present study investigates the association between oral microbiome diversity and all-cause mortality in hypertensive individuals, with rigorous adjustment for potential confounders. Leveraging oral microbiome diversity data and mortality record from NHANES, this study examines whether greater alpha diversity correlates with improved survival outcomes. By elucidating the role of oral microbiome diversity in mortality risk, this research may inform strategies for managing hypertension and its complications.

## Materials and methods

### Study population

The baseline data were obtained from NHANES, a continuous cross-sectional survey designed with a nationally representative, multi-stage sampling approach. NHANES systemically evaluates the health status, disease burdens and nutritional patterns of the non-institutionalised U.S civilians through standardised protocols encompassing demographic interviews, dietary assessments, physical examinations, laboratory analyses, and comprehensive medical histories. The study protocols received approval from the Institutional Review Board of the Centres for Disease Control and Prevention (CDC), with written informed consent from all participants prior to data collection [26].

**Figure 1.**
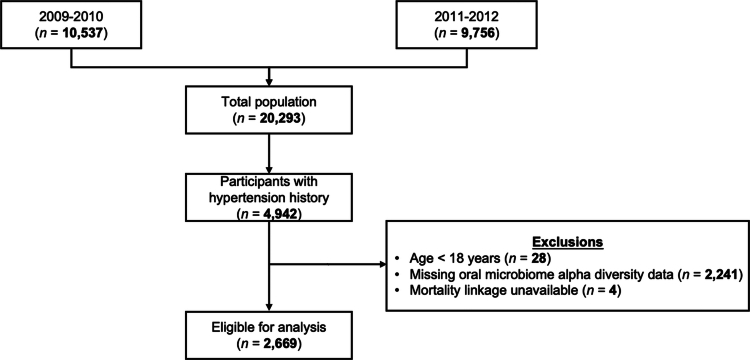
Flow diagram showing the inclusion and exclusion criteria applied to participants from the National Health and Nutrition Examination Survey (NHANES) 2009–2012 cycles. Individuals were selected based on available oral microbiome data, hypertension status, and mortality follow-up.

Our study adhered to the Strengthening the Reporting of Observational Studies in Epidemiology (STROBE) guidelines. From a total of 20,293 participants across two consecutive NHANES cycles (2009–2010 and 2011–2012), we first selected 4,942 individuals who had ever been told by a doctor or other health professional that they had hypertension. We then excluded participants who were under 18 years old (*N* = 28), those without oral microbiome *α*-diversity data (*N* = 2,241), and those with missing follow-up data (*N* = 4). After applying these criteria, the final analytic sample consisted of 2,669 participants (Figure 1).

### Oral microbiome diversity evaluation

The analytic approaches and results of oral microbiome diversity were obtained from the NHANES Oral Microbiome Project conducted on participants aged 14−69 during the 2009−2010 and 2011−2012 cycles [27]. Briefly, an oral rinse sample was collected from each participant by having them rinse with mouthwash for 5 seconds, followed by three 5-second gargles. The examiner carefully retrieved the oral rinse samples which were processed for DNA extraction with the Puregene DNA purification kit. The purified DNA was stored at −20 °C until processing. Polymerase chain reaction (PCR) amplification and sequencing were performed according to the published protocols and standards from the Earth Microbiome Project [28].

The DADA2 pipeline (v. 1.2.1) was applied to identify amplicon sequence variants (ASVs), representing unique microbial sequences after quality control. These ASVs were stored in a feature table with read counts per sample. Additionally, DADA2 was used to construct a phylogenetic tree based on ASV data. Subsequent analysis identified a non-bacterial ASV (SV1032) associated with clustering. To address this, the ASV feature table excluding SV1032 was created for further analysis.

Microbiome diversity was evaluated using both *α*- and *β*-diversity metrics following the NHANES oral microbiome processing pipeline implemented in QIIME (v1.9.1) [29]. For *α*-diversity, four indices were calculated on rarefied ASV tables (10,000 reads per sample): Simpson index, Shannon–Weiner index, Faith’s phylogenetic diversity, and observed ASVs. Observed ASVs and Faith’s phylogenetic diversity measured microbial richness, whereas the Shannon–Weiner and Simpson indices captured both richness and evenness [30,31]. For *β*-diversity, pairwise dissimilarity matrices were generated using Bray–Curtis, unweighted UniFrac, and weighted UniFrac distance metrics. Principal coordinate analysis (PCoA) was then performed for each distance matrix, and the first four principal coordinates (PCoA1–PCoA4) were extracted to represent the major axes of between-sample microbial variation for downstream regression analyses.

### Assessment of hypertension

Blood pressure was measured three times after participants had been seated quietly for at least five minutes, and the mean value was used for analysis. Hypertension was defined as systolic blood pressure ≥ 140 mm Hg, diastolic blood pressure ≥ 90 mm Hg, self-reported physician diagnosis, or current use of antihypertensive medication [32,33].

### Outcome ascertainment

The endpoint was all-cause mortality. Mortality data were sourced from the publicly available NHANES Linked Mortality File, which tracked participants’ survival status and causes of death using matched respondent sequence numbers, covering data up to December 31, 2019.

### Assessment of covariates

The selection of covariates was based on existing literature reporting the relationship between oral health and mortality [34]. To further ensure appropriate adjustment, we also constructed a directed acyclic graph (DAG) to inform the identification of potential confounders included in the multivariable models (Fig. S1). Covariate data were primarily collected at baseline (2009–2010 and 2011–2012 cycles) through structured questionnaires [26]. Sociodemographic characteristics included age, sex (male or female), ethnicity (Mexican American, non-Hispanic White, non-Hispanic Black, or others), income level (household income-to-poverty ratio as a proxy for socioeconomic status: <1.01, 1.01–3.01, ≥3.01) [35], highest educational attainment (high school or below, or college or above) [34], and marital status (married/partnered or other). Lifestyle factors included smoking status (never, former, or current smoker), drinking status (never, former, mild, moderate or heavy) [36], and body mass index (BMI: <25.0 kg/m², 25.0–29.9 kg/m², or ≥30.0 kg/m²) [37]. Comorbidities including diabetes, cancer, coronary heart disease, congestive heart failure, angina, myocardial infarction, stroke, liver disease, and thyroid disease were identified based on participants’ self-reported information. For statistical adjustments, three binary variables were created to categorise the comorbidities. Participants were classified as having CVDs if they had at least one of the following conditions: angina, coronary heart disease, congestive heart failure, myocardial infarction, or stroke. Similarly, individuals with a history of diabetes, liver disease, or thyroid disease were categorised under metabolic-related diseases, and those with a history of cancer were classified as having cancer [34]. Additionally, participants underwent clinical periodontal examinations conducted by trained and calibrated dentists, in which the severity of periodontitis was determined by the Centres for Disease Control and Prevention (CDC) and American Academy of Periodontology (AAP) case definitions [38]. To minimise potential bias associated with the high prevalence of mild periodontitis in the population, participants with no or mild periodontitis were grouped together, while those with moderate or severe periodontitis were categorised as the other group [39].

### Statistical analysis

Baseline characteristics were summarised for all participants stratified by the quartiles of oral microbiome diversity using the Simpson index. Continuous variables were presented as mean (standard deviation, SD) while categorical variables were expressed as count (percentage). Group differences were assessed using analysis of variance for continuous variables and the Rao–Scott χ² test for categorical variables. Additionally, Pearson correlation analysis was performed to evaluate the relationships among the four microbial diversity indices.

Given the complex multistage cluster survey design of NHANES, Mobile Examination Centre exam sampling weights were applied following CDC recommendations [40]. Then, oral microbiome diversity indices were categorised into quartiles to create four groups based on the distribution. Kaplan–Meier survival curves were generated to depict survival probabilities over time across groups based on oral microbiome diversity, and group differences were assessed using the log-rank test. Survival curves were presented with 95% confidence intervals, along with risk tables showing the number of participants at risk at each time point.

To estimate the association between oral microbiome diversity and all-cause mortality, weighted Cox proportional hazards models with age as the time scale (left-truncated at participants’ age at examination) were fitted using the *survey* and *survival* packages. The lowest diversity group (first quartile) was used as the reference category. Hazard ratios (HRs) and 95% confidence intervals (CIs) were estimated across three progressively adjusted models: Model 1 (MV1) adjusted for sex; Model 2 (MV2) further included BMI, ethnicity, income, education, marital status, smoking and drinking status; and Model 3 (MV3) additionally adjusted for periodontitis and comorbidities including CVDs, metabolic-related diseases, and cancer.A *P*-value for trend (*P*_trend_) was calculated to assess linear trends across quartiles. In addition, HRs per one–standard deviation (1-SD) increment in each diversity index were calculated to evaluate associations on a continuous scale. Similarly, *β*-diversity measures represented by the first four principal coordinates (PCoA1–PCoA4) from Bray–Curtis, unweighted UniFrac, and weighted UniFrac analyses were examined using the same weighted Cox regression models to assess associations with all-cause mortality. To assess the validity of the Cox model, we tested the proportional hazards assumption using Schoenfeld residuals [41] and examined multicollinearity among predictor variables using the adjusted Generalised Variance Inflation Factor [42].

Subsequently, restricted cubic splines (RCS) were used to model microbiome diversity as a continuous variable, allowing for a flexible evaluation of potential nonlinear associations with mortality risk. The first quartile cut-off value was selected as the reference point, and the model was constructed with three knots placed at the 10th, 50th, and 90th percentiles of the diversity distribution.

Furthermore, interaction and stratified analyses were conducted to evaluate whether the associations between alpha diversity indices and mortality risk differed by sex and BMI.

Sensitivity analyses were performed to confirm the robustness of the findings. Multiple imputation (MI) was conducted using the *mice* package to handle missing data by generating five imputed datasets, and the results were combined using Rubin’s rules [43]. Moreover, we excluded individuals with CVDs, metabolic-related diseases or cancer and recalculated the association estimates in the sensitive analyses.

All statistical analyses were conducted using R version 4.4.1. All statistical tests were two-sided, and *P* < 0.05 was considered statistically significant.

## Results

### Baseline characteristics

The baseline characteristics were summarised in Table 1. This analytical cohort comprised 2,669 participants with a mean age of 51.87 ± 12.53 years, including 47.73% females. A significant proportion reported having systemic conditions, including CVDs (14.57%), metabolic-related diseases (32.00%), and cancer (9.32%). Among those who underwent periodontal assessments, 54.48% had moderate or severe periodontitis. Participants with higher oral microbial diversity were more likely to be younger, male, Mexican American or non-Hispanic White, and to have moderate-to-severe periodontitis. In contrast, lower microbial diversity was more prevalent among non-Hispanic White and cancer participants (*P* < 0.05). Pearson correlation analysis showed a high correlation between the Simpson and Shannon–Weiner indices, as well as between observed ASVs and Faith’s PD (Fig. S2).

**Table 1.** Baseline characteristics of the subjects.

		Oral microbiome diversity				
Variables	Overall	Quartile1	Quartile2	Quartile3	Quartile4	*P*-Value
	(*N* = 2669)	(*N* = 668)	(*N* = 667)	(*N* = 667)	(*N* = 667)	
Age, Mean ± SD	51.87 ± 12.53	52.08 ± 12.89	52.36 ± 12.67	52.41 ± 12.09	50.64 ± 12.41	0.009
**Sex, *n* (%)**						<0.001
Female	1,274 (47.73)	356 (53.29)	326 (48.88)	313 (46.93)	279 (41.83)	
Male	1,395 (52.27)	312 (46.71)	341 (51.12)	354 (53.07)	388 (58.17)	
**Ethnicity (%)**						0.014
Mexican American	391 (14.65)	76 (11.38)	90 (13.49)	114 (17.09)	111 (16.64)	
Non-Hispanic Black	870 (32.60)	226 (33.83)	198 (29.69)	218 (32.68)	228 (34.18)	
Non-Hispanic White	916 (34.32)	246 (36.83)	253 (37.93)	215 (32.23)	202 (30.28)	
Others	492 (18.43)	120 (17.96)	126 (18.89)	120 (17.99)	126 (18.89)	
**Education, *n* (%)**						0.170
High school or below	1,398 (52.44)	358 (53.67)	350 (52.63)	326 (48.88)	364 (54.57)	
College or above	1,268 (47.56)	309 (46.33)	315 (47.37)	341 (51.12)	303 (45.43)	
Marital status, *n* (%)						0.057
Married/living with partner	1,561 (59.29)	377 (57.12)	370 (56.49)	398 (60.58)	416 (62.93)	
Others	1,072 (40.71)	283 (42.88)	285 (43.51)	259 (39.42)	245 (37.07)	
**Ratio of family income to poverty, *n* (%)**						0.291
<1.01	620 (25.20)	155 (24.92)	174 (28.20)	134 (22.30)	157 (25.32)	
1.01–3.01	975 (39.63)	241 (38.75)	233 (37.76)	244 (40.60)	257 (41.45)	
≥3.01	865 (35.16)	226 (36.33)	210 (34.04)	223 (37.10)	206 (33.23)	
**Smoking status, *n* (%)**						0.159
Never	1,272 (51.04)	308 (49.44)	308 (49.52)	338 (54.34)	318 (50.88)	
Former	648 (26.00)	179 (28.73)	170 (27.33)	152 (24.44)	147 (23.52)	
Current	572 (22.95)	136 (21.83)	144 (23.15)	132 (21.22)	160 (25.60)	
**Drinking status, *n* (%)**						0.178
Never	285 (12.18)	78 (13.40)	78 (13.18)	64 (11.05)	65 (11.07)	
Former	515 (22.01)	136 (23.37)	124 (20.95)	129 (22.28)	126 (21.47)	
Mild	726 (31.03)	185 (31.79)	175 (29.56)	201 (34.72)	165 (28.11)	
Moderate	336 (14.36)	79 (13.57)	93 (15.71)	73 (12.61)	91 (15.50)	
Heavy	478 (20.43)	104 (17.87)	122 (20.61)	112 (19.34)	140 (23.85)	
**BMI, *n* (%)**						0.323
<25 kg/m2	427 (16.20)	122 (18.63)	105 (15.98)	102 (15.41)	98 (14.80)	
25.0–29.9 kg/m2	801 (30.39)	192 (29.31)	216 (32.88)	194 (29.31)	199 (30.06)	
≥30.0 kg/m2	1,408 (53.41)	341 (52.06)	336 (51.14)	366 (55.29)	365 (55.14)	
Cardiovascular diseases, *n* (%)	384 (14.57)	111 (16.82)	104 (15.85)	90 (13.68)	79 (11.95)	0.055
Metabolic-Related Diseases, *n* (%)	854 (32.00)	230 (34.43)	206 (30.88)	220 (32.98)	198 (29.69)	0.247
Cancer, *n* (%)	245 (9.32)	78 (11.82)	73 (11.18)	50 (7.62)	44 (6.66)	0.002
**Periodontitis status, *n* (%)**						<0.001
No/mild	935 (45.52)	244 (51.80)	246 (48.81)	238 (44.82)	207 (37.77)	
Moderate/severe	1,119 (54.48)	227 (48.20)	258 (51.19)	293 (55.18)	341 (62.23)	

### Associations relationships between oral microbiome diversity and all-cause mortality

**Figure 2.**
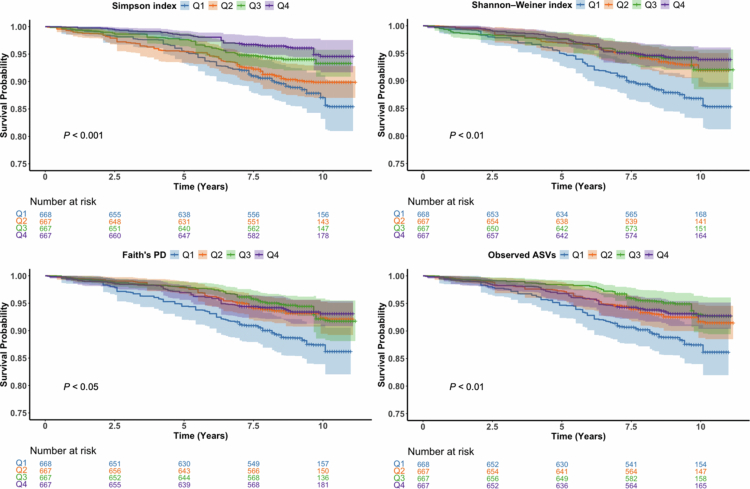
Kaplan–Meier survival curves illustrating all-cause mortality across quartiles of oral microbiome diversity, assessed by four alpha-diversity indices: observed ASVs, Faith’s Phylogenetic Diversity (PD), Shannon–Weiner index, and Simpson index. Log-rank tests were used to evaluate survival differences across quartiles for each index.

During a total follow-up of 22,993 person-years (mean = 8.61 years), 268 deaths were recorded, including 83 from CVDs, 76 from cancer, 101 from other diseases, and 8 from accidental injuries (Table S1). To further examine group differences in survival, Kaplan–Meier curves were generated. As illustrated in Figure 2, participants with higher oral microbial diversity consistently exhibited significantly better survival probabilities across all *α*-diversity indices (Shannon–Weiner index, Simpson index, Faith’s PD, and observed ASVs). All log-rank tests were statistically significant (*p* < 0.05).

**Figure 3.**
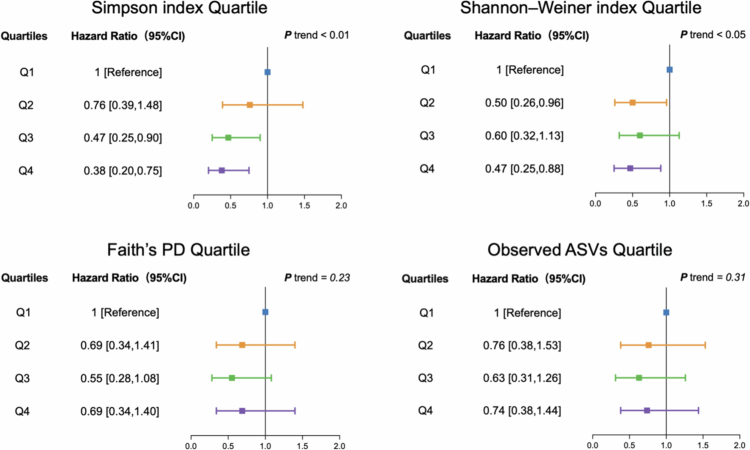
Forest plot of multivariable-adjusted hazard ratios (HRs) and 95% confidence intervals for all-cause mortality across quartiles of oral microbiome diversity. Models adjust for sex, ethnicity, income, education, marital status, smoking status, drinking status, BMI, periodontal disease, and comorbidities (cardiovascular diseases, metabolic-related diseases, and cancer), with age handled via left truncation.

In the weighted Cox regression model, we observed significant inverse associations between oral microbiome diversity, assessed by the Simpson and Shannon–Weiner indices, and all-cause mortality (Table 2 and Figure 3). In the fully adjusted model, individuals in the highest quartile (Q4) of diversity had substantially lower mortality risk compared to those in the lowest quartile (Q1). Specifically, the HR was 0.38 (95% CI: 0.20–0.75, *P*_trend_ < 0.01) for the Simpson index and 0.47 (95% CI: 0.25–0.88, *P*_trend_ < 0.05) for the Shannon index, corresponding to an approximately 62% and 53% reduction in mortality risk, respectively. For analyses using continuous measures, each one–standard deviation (1-SD) increase in diversity was associated with HRs of 0.84 (95% CI: 0.73–0.97) for the Simpson index and 0.75 (95% CI: 0.60–0.93) for the Shannon index. In contrast, the associations for Observed ASVs and Faith’s PD were not statistically significant. The weighted Cox regression models were validated by Schoenfeld residuals, which supported the proportional hazards assumption (Fig. S3), and no significant multicollinearity was detected based on the adjusted generalised variance inflation factor (Table S2).

**Table 2.** Association between oral microbiome diversity indices and all-cause mortality.

Oral microbiome diversity	Median (IQR)	Deaths/person-years	Model 1	Model 2	Model 3
			HR (95% CI)	HR (95% CI)	HR (95% CI)
**Simpson index**					
Q1	0.85(0.81−0.87)	84/5734	Ref.	Ref.	Ref.
Q2	0.90(0.89−0.91)	80/5637	0.76 [0.49−1.16]	0.85 [0.53−1.36]	0.76 [0.39−1.48]
Q3	0.93(0.92−0.93)	61/5732	0.49 [0.31−0.79]	0.58 [0.35−0.98]	0.47 [0.25−0.90]
Q4	0.95(0.94−0.96)	43/5890	0.36 [0.21−0.61]	0.38 [0.21−0.68]	0.38 [0.20−0.75]
Per SD-unit increment			0.82 [0.73−0.92]	0.87 [0.77−0.99]	0.84 [0.73−0.97]
*p* trend			<0.001	<0.001	<0.01
**Shannon–Weiner index**					
Q1	3.82(3.44−4.04)	92/5754	Ref.	Ref.	Ref.
Q2	4.41(4.30−4.53)	68/5654	0.60 [0.38−0.95]	0.61 [0.37−1.03]	0.50 [0.26−0.96]
Q3	4.82(4.74−4.93)	55/5759	0.58 [0.36−0.92]	0.65 [0.38−1.10]	0.60 [0.32−1.13]
Q4	5.30(5.17−5.55)	53/5826	0.52 [0.33−0.83]	0.50 [0.30−0.85]	0.47 [0.25−0.88]
Per SD-unit increment			0.73 [0.62−0.86]	0.79 [0.67−0.93]	0.75 [0.60−0.93]
*p* trend			<0.01	<0.05	<0.05
**Faith's PD**					
Q1	10.27(9.09−11.09)	89/5668	Ref.	Ref.	Ref.
Q2	12.99(12.45−13.49)	64/5733	0.66 [0.41−1.05]	0.67 [0.39−1.14]	0.69 [0.34−1.41]
Q3	15.22(14.59−15.86)	58/5760	0.62 [0.39−0.96]	0.57 [0.35−0.95]	0.55 [0.28−1.08]
Q4	18.33(17.27−19.79)	57/5831	0.74 [0.46−1.19]	0.63 [0.38−1.05]	0.69 [0.34−1.40]
Per SD-unit increment			0.85 [0.70−1.04]	0.85 [0.70−1.03]	0.87 [0.66−1.14]
*p* trend			0.12	<0.05	0.23
**Observed ASVs**					
Q1	75.65(61.50−86.40)	90/5642	Ref.	Ref.	Ref.
Q2	109.20(101.80−116.45)	68/5718	0.68 [0.43−1.07]	0.81 [0.47−1.38]	0.76 [0.38−1.53]
Q3	138.20(130.15−145.30)	51/5843	0.56 [0.34−0.91]	0.61 [0.35−1.04]	0.63 [0.31−1.26]
Q4	178.60(165.45−197.15)	59/5790	0.74 [0.47−1.18]	0.72 [0.43−1.19]	0.74 [0.38−1.44]
Per SD-unit increment			0.83 [0.68−1.02]	0.79 [0.67−0.93]	0.87 [0.67−1.14]
*p* trend			0.09	0.09	0.31

Using principal coordinates (PCoA1–PCoA4) as *β*-diversity indicators, two significant associations with all-cause mortality were identified in the fully adjusted model: Bray–Curtis PCoA2 (HR = 0.71, 95% CI: 0.58–0.88) and weighted UniFrac PCoA1 (HR = 0.76, 95% CI: 0.59–0.99) (Table S3).

**Figure 4.**
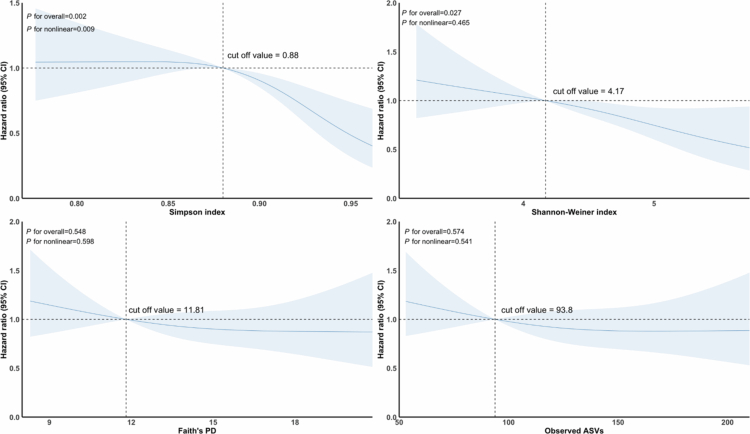
Restricted cubic spline (RCS) curves showing the association between oral microbiome diversity and all-cause mortality in hypertensive individuals. Models adjust for sex, ethnicity, income, education, marital status, smoking status, drinking status, BMI, periodontal disease, and comorbidities (cardiovascular diseases, metabolic-related diseases, and cancer), with age handled via left truncation.

RCS analysis was applied to evaluate the potential nonlinearity of the associations between oral microbiome diversity and mortality. As shown in Figure 4, a significant nonlinear relationship was observed for the Simpson index (*P*_nonlinear_ < 0.01), with mortality risk remaining relatively stable initially, but beginning to decline sharply once the Simpson index exceeded approximately 0.86, forming an inverted L-shaped curve. On the contrary, no statistically significant nonlinear associations were observed between the Shannon-Weiner index, observed ASVs, or Faith’s PD and mortality.

### Interaction effects in mortality

Interaction analysis (Table S4) revealed a statistically significant interaction between sex and the Shannon–Weiner index (*P* < 0.05) regarding mortality risk, whereas no significant interaction was observed for the Simpson index. Subsequently, sex-stratified analyses were conducted for both the Shannon–Weiner and Simpson indices (Fig. S4). In the fully adjusted model, a pronounced sex-specific pattern was observed. Among women, higher oral microbiome diversity was significantly associated with lower all-cause mortality. Specifically, participants in the Q4 had markedly lower mortality risk compared with those in the Q1, with HRs of 0.34 (95% CI: 0.14–0.83, *P*_trend_ < 0.05) for the Shannon–Weiner index and 0.18 (95% CI: 0.06–0.53, *P*_trend_ < 0.01) for the Simpson index. In contrast, no statistically significant associations were found among men. For continuous analyses, each one–standard deviation (1-SD) increase in diversity was associated with HRs of 0.53 (95% CI: 0.37–0.76) for the Shannon–Weiner index and 0.62 (95% CI: 0.51–0.76) for the Simpson index in women, whereas no significant associations were observed in men (Table. S5).

### Sensitivity analysis

Sensitivity analyses were conducted to assess the robustness of the findings. After sequentially excluding participants with cancer, CVDs, and metabolic-related conditions, the associations between oral microbiome diversity and all-cause mortality remained consistent with the main results (Table S6). Additionally, results from weighted Cox regression analyses based on multiply imputed datasets also aligned with the primary findings (Table S7), further confirming the robustness of the observed associations.

## Discussion

In this study, we found that greater oral microbiome diversity was significantly associated with lower all-cause mortality among individuals with hypertension. Specifically, higher *α*-diversity, as measured by the Shannon–Weiner and Simpson indices, predicted reduced mortality risk, while *β*-diversity analyses identified two significant components (Bray–Curtis PCoA2 and weighted UniFrac PCoA1) showing inverse associations with mortality. Moreover, interaction modelling revealed a significant sex-specific effect for the Shannon–Weiner index, and stratified analyses further demonstrated that higher diversity was associated with lower mortality risk among women but not among men. Taken together, these findings suggest that greater oral microbial diversity is linked to lower mortality risk in hypertensive individuals.

Our analysis revealed a significant inverse association between oral microbiome *α*-diversity and all-cause mortality among hypertensive individuals, persisting after comprehensive adjustment for potential confounders. This finding aligns with previous NHANES investigations in general populations [18,44], yet suggests critical metric-specific divergences. In our study, all four *α*-diversity metrics showed inverse associations with mortality risk, but only the associations for Shannon–Weiner and Simpson indices reached statistical significance, whereas Faith’s PD and observed ASVs did not reach statistical significance. This discrepancy, in contrast to general population studies where all four *α*-diversity metrics were inversely associated with mortality [18,44], may be partially attributed to factors such as smaller sample size, differences in within-group variance, or the inherent ecological properties of the metrics. Notably, the Shannon–Weiner and Simpson indices integrate both microbial richness (species count) and evenness (abundance distribution), whereas Faith’s PD and ASV richness reflect richness alone [45]. Beyond these within-individual diversity measures, we further examined between-individual variation in microbial community composition. *β*-diversity analyses supported the *α*-diversity findings, as Bray–Curtis PCoA2 and weighted UniFrac PCoA1 were significantly associated with all-cause mortality. This suggests that not only overall diversity but also community composition of the oral microbiome may influence survival among hypertensive individuals.

Within the hypertensive population, variations in oral microbial diversity may act synergistically with hypertension-related systemic conditions such as inflammation and vascular dysfunction [21], thereby influencing long-term survival. Maintaining ecological stability within the oral microbiome may help preserve host–microbe homoeostasis, preventing the dominance of pathogenic taxa or the loss of beneficial commensals [46]. Conversely, disruption of this balance may exacerbate the inflammatory and vascular burden already present in hypertensive individuals [47], thereby contributing to increased mortality risk.

Interaction modelling revealed a significant sex-specific effect for the Shannon–Weiner index, with stratified analyses indicating a stronger inverse association between oral microbiome diversity and mortality risk among women. This sex-specific association aligns with well-documented sex differences in cardiovascular diseases, suggesting potential biological relevance. This may reflect sex-specific physiological mechanisms, such as oestrogen-mediated modulation of vascular function or differential immune responses influencing microbial–host interactions [48–50]. Although sex hormone levels were not measured in this study, these mechanistic explanations remain hypothetical. Therefore, the observed sex difference should be interpreted with caution, and replication in independent cohorts is necessary. Future studies are needed to explore microbiome-related cardiovascular risk through a sex-specific lens, particularly focusing on hormonal and immunological pathways.

Interestingly, we found a positive association between periodontitis and oral microbiome diversity, consistent with prior studies [51,52]. However, periodontitis has also been linked to increased mortality [34], suggesting that diversity itself may not mediate this relationship and highlighting the need for further investigation [44], further emphasising the complexity of the relationship between oral health and systemic outcomes.

One of the main strengths of our study lies in its focus on a hypertensive population, which enabled us to specifically examine the prognostic relevance of oral microbiome diversity in individuals already at elevated cardiovascular risk. Building on this targeted design, we conducted extensive sensitivity analyses to confirm the robustness of our findings, applying multiple statistical strategies to handle missing data, control for potential confounders, and evaluate possible interactions between oral microbiome diversity and other variables. Finally, the use of NHANES, a nationally representative dataset with rigorous data collection protocols, further enhances the reliability and generalisability of our results.

Despite these strengths, several limitations should be acknowledged. First, oral microbiome data were obtained from a single mouthwash sample, potentially underrepresenting the diversity across distinct oral ecological niches such as the subgingival area or tongue dorsum. Moreover, our analysis focused on overall diversity metrics rather than specific microbial taxa, functional pathways, or ecological guilds, which provides only a coarse summary of the microbial community and limits the identification of specific microbial drivers of mortality risk. Second, the cross-sectional nature of microbiome assessment precludes evaluation of temporal variation and its dynamic relationship with health outcomes. Thus, causal inference cannot be established. Third, lifestyle factors such as diet and physical activity, which can influence both oral microbiome composition and health outcomes, were not assessed and may confound the observed associations. Fourth, while comprehensive covariate adjustment was performed, residual confounding cannot be fully excluded. Because this study was conducted exclusively among individuals with hypertension, the findings should be interpreted within this population and may not be generalisable to individuals without hypertension.

## Conclusion

In conclusion, among individuals with hypertension, greater oral microbiome diversity was associated with lower all-cause mortality risk. Higher *α*-diversity (Shannon–Weiner and Simpson indices) and several *β*-diversity principal components showed consistent inverse associations. Moreover, a significant sex-specific pattern was observed, with stronger associations among women. These findings suggest that oral microbial diversity may serve as a potential ecological indicator of survival in hypertensive populations, highlighting the need for further research to clarify underlying mechanisms.

## Supplementary Material

Supplementary materials.docx Supplementary Materials.docx

## Data Availability

The datasets analysed during the current study are accessible from the NHANES database (https://www.cdc.gov/nchs/nhanes/index.htm).

## References

[1] Wong CC, Yu J. Gut microbiota in colorectal cancer development and therapy. Nat Rev Clin Oncol. 2023;20(7):429–452. doi: 10.1038/s41571-023-00766-x 37169888

[2] Witkowski M, Weeks TL, Hazen SL. Gut microbiota and cardiovascular disease. Circ Res. 2020;127(4):553–570. doi: 10.1161/CIRCRESAHA.120.316242 32762536 PMC7416843

[3] Dabke K, Hendrick G, Devkota S. The gut microbiome and metabolic syndrome. J Clin Invest. 2019;129(10):4050–4057. doi: 10.1172/JCI129194 31573550 PMC6763239

[4] Rajasekaran JJ, Krishnamurthy HK, Bosco J, et al. Oral microbiome: a review of its impact on oral and systemic health. Microorganisms. 2024;12(9):1797. doi: 10.3390/microorganisms12091797 39338471 PMC11434369

[5] Da D, Ge S, Zhang H, et al. Risk factors and related oral microbes of periodontitis. Int Dent J. 2024;74:S398–S399. doi: 10.1016/j.identj.2024.07.1224

[6] Liu R, Liu Y, Yi J, et al. Imbalance of oral microbiome homeostasis: the relationship between microbiota and the occurrence of dental caries. BMC Microbiol. 2025;25(1):46. doi: 10.1186/s12866-025-03762-6 39865249 PMC11770982

[7] Tonelli A, Lumngwena EN, Ntusi NAB. The oral microbiome in the pathophysiology of cardiovascular disease. Nat Rev Cardiol. 2023;20(6):386–403. doi: 10.1038/s41569-022-00825-3 36624275

[8] Long J, Cai Q, Steinwandel M, et al. Association of oral microbiome with type 2 diabetes risk. J Periodontal Res. 2017;52(3):636–643. doi: 10.1111/jre.12432 28177125 PMC5403709

[9] Esberg A, Johansson L, Johansson I, et al. Oral Microbiota identifies patients in early onset rheumatoid arthritis. Microorganisms. 2021;9(8):1657. doi: 10.3390/microorganisms9081657 34442739 PMC8400434

[10] Jungbauer G, Stähli A, Zhu X, et al. Periodontal microorganisms and alzheimer disease - a causative relationship? Periodontol 2000. 2022;89(1):59–82. doi: 10.1111/prd.12429 35244967 PMC9314828

[11] Irfan M, Delgado RZR, Frias-Lopez J. The oral microbiome and cancer. Front Immunol. 2020;11:591088. doi: 10.3389/fimmu.2020.591088 33193429 PMC7645040

[12] He J, Li Y, Cao Y, et al. The oral microbiome diversity and its relation to human diseases. Folia Microbiol (Praha). 2015;60(1):69–80. doi: 10.1007/s12223-014-0342-2 25147055

[13] Koren O, Spor A, Felin J, et al. Human oral, gut, and plaque microbiota in patients with atherosclerosis. Proc Natl Acad Sci. 2011;108(supplement_1):4592–4598. doi: 10.1073/pnas.1011383107 20937873 PMC3063583

[14] Singh H, Torralba MG, Moncera KJ, et al. Gastro-intestinal and oral microbiome signatures associated with healthy aging. GeroScience. 2019;41(6):907–921. doi: 10.1007/s11357-019-00098-8 31620923 PMC6925087

[15] Zheng L, Yang R, Li R, et al. Exploring the association between thyroid function and oral microbiome diversity: an NHANES analysis. J Endocr Soc. 2023;7(11):bvad125. doi: 10.1210/jendso/bvad125 37818404 PMC10561013

[16] Hosgood HD, Cai Q, Hua X, et al. Variation in oral microbiome is associated with future risk of lung cancer among never-smokers. Thorax. 2021;76(3):256–263. doi: 10.1136/thoraxjnl-2020-215542 33318237 PMC8513501

[17] Yuan X, Wu J, Chen R, et al. Characterization of the oral microbiome of children with type 1 diabetes in the acute and chronic phases. J Oral Microbiol. 2022;14(1):2094048. doi: 10.1080/20002297.2022.2094048 35859767 PMC9291685

[18] Shen J, Chen H, Zhou X, et al. Oral microbiome diversity and diet quality in relation to mortality. J Clin Periodontol. 2024;51(11):1478–1489. doi: 10.1111/jcpe.14050 39188084

[19] Mondal R, Ritu RB, Kitaoka K, et al. Oral microbiome alpha diversity and all-cause, cardiovascular, and non-cardiovascular mortality in US adults: evidence from the NHANES 2009-2019. Atherosclerosis. 2025;401:119074. doi: 10.1016/j.atherosclerosis.2024.119074 39644613 PMC12425798

[20] Rethy L, Shah NS, Paparello JJ, et al. Trends in hypertension-related cardiovascular mortality in the United States, 2000 to 2018. Hypertension. 2020;76(3):e23–e25. doi: 10.1161/HYPERTENSIONAHA.120.15153 32654559 PMC9390965

[21] Gallo G, Volpe M, Savoia C. Endothelial dysfunction in hypertension: current concepts and clinical implications. Front Med (Lausanne). 2021;8:798958. doi: 10.3389/fmed.2021.798958 35127755 PMC8811286

[22] Virdis A, Dell’Agnello U, Taddei S. Impact of inflammation on vascular disease in hypertension. Maturitas. 2014;78(3):179–183. doi: 10.1016/j.maturitas.2014.04.012 24846805

[23] Peng X, Cheng L, You Y, et al. Oral microbiota in human systematic diseases. Int J Oral Sci. 2022;14(1):14. doi: 10.1038/s41368-022-00163-7 35236828 PMC8891310

[24] Li Q, Ouyang X, Lin J. The impact of periodontitis on vascular endothelial dysfunction. Front Cell Infect Microbiol. 2022;12:998313. doi: 10.3389/fcimb.2022.998313 36118034 PMC9480849

[25] Zenobia C, Darveau RP. Does oral endotoxin contribute to systemic inflammation? Front Oral Health. 2022;3:911420. doi: 10.3389/froh.2022.911420 35677024 PMC9169450

[26] National Health and Nutrition Examination Survey. https://www.cdc.gov/nchs/nhanes/index.htm

[27] NHANES 2009-2012 Oral Microbiome. https://wwwn.cdc.gov/Nchs/Nhanes/Omp/Default.aspx

[28] 16S Illumina Amplicon Protocol. https://earthmicrobiome.org/protocols-and-standards/16s/

[29] Bolyen E, Rideout JR, Dillon MR, et al. Reproducible, interactive, scalable and extensible microbiome data science using QIIME 2. NatBi. 2019;37(8):852–857. doi: 10.1038/s41587-019-0209-9 PMC7015180 31341288

[30] Simpson EH. Measurement of diversity. Natur. 1949;163(4148):688–688. doi: 10.1038/163688a0

[31] Shannon CE. A mathematical theory of communication. The Bell System Technical Journal. 1948;27(3):379–423. doi: 10.1002/j.1538-7305.1948.tb01338.x

[32] Cao Y, Li P, Zhang Y, et al. Association of systemic immune inflammatory index with all-cause and cause-specific mortality in hypertensive individuals: results from NHANES. Front Immunol. 2023;14:1087345. doi: 10.3389/fimmu.2023.1087345 36817427 PMC9932782

[33] Li C, Zhang Z, Luo X, et al. The triglyceride-glucose index and its obesity-related derivatives as predictors of all-cause and cardiovascular mortality in hypertensive patients: insights from NHANES data with machine learning analysis. Cardiovasc Diabetol. 2025;24(1):47. doi: 10.1186/s12933-025-02591-1 39881352 PMC11780913

[34] Larvin H, Baptiste PJ, Gao C, et al. All-cause and cause-specific mortality in US adults with periodontal diseases: a prospective cohort study. J Clin Periodontol. 2024;51(9):1157–1167. doi: 10.1111/jcpe.14002 38802320

[35] Washington DUSDoHHS. Poverty guidelines, research, and measurement. 2012.

[36] Chen Q, Ge R, Wu Y, et al. The associations of coffee consumption, coffee types, and caffeine metabolites with periodontitis: results from NHANES 2009-2014. J Periodontol. 2024;95(8):778–788. doi: 10.1002/JPER.23-0322 37815812

[37] Obesity and overweight. https://www.who.int/news-room/fact-sheets/detail/obesity-and-overweight

[38] Eke PI, Dye BA, Wei L, et al. Update on prevalence of periodontitis in adults in the United States: NHANES 2009 to 2012. J Periodontol. 2015;86(5):611–622. doi: 10.1902/jop.2015.140520 25688694 PMC4460825

[39] Li W, Peng J, Shang Q, et al. Periodontitis and the risk of all-cause and cause-specific mortality among US adults with diabetes: a population-based cohort study. J Clin Periodontol. 2024;51(3):288–298. doi: 10.1111/jcpe.13901 37967814

[40] Weighting. https://wwwn.cdc.gov/nchs/nhanes/tutorials/weighting.aspx?utm_source=chatgpt.com

[41] Schoenfeld D. Partial residuals for the proportional hazards regression model. Biometrika. 1982;69(1):239–241. doi: 10.1093/biomet/69.1.239

[42] Shrestha N. Detecting Multicollinearity in regression analysis. American Journal of Applied Mathematics and Statistics. 2020;8(2):39–42. doi: 10.12691/ajams-8-2-1

[43] van Buuren S, Groothuis-Oudshoorn K. Mice: multivariate imputation by chained equations in R. J Stat Softw. 2011;45(3):1–67. doi: 10.18637/jss.v045.i03

[44] Yang Z, He F, Huang H, et al. Association of oral microbiome diversity and all-cause mortality in the general US population and in individuals with chronic diseases: a prospective cohort study. J Clin Periodontol. 2024;51(11):1490–1501. doi: 10.1111/jcpe.14056 39152675

[45] Kers JG, Saccenti E. The power of microbiome studies: some considerations on which alpha and beta metrics to use and how to report results. Front Microbiol. 2022;12:2021. doi: 10.3389/fmicb.2021.796025 PMC8928147 35310396

[46] Kilian M, Chapple IL, Hannig M, et al. The oral microbiome - an update for oral healthcare professionals. Br Dent J. 2016;221(10):657–666. doi: 10.1038/sj.bdj.2016.865 27857087

[47] Kleinstein SE, Nelson KE, Freire M. Inflammatory networks linking oral microbiome with systemic health and disease. J Dent Res. 2020;99(10):1131–1139. doi: 10.1177/0022034520926126 32459164 PMC7443998

[48] Baker JM, Al-Nakkash L, Herbst-Kralovetz MM. Estrogen−gut microbiome axis: physiological and clinical implications. Maturitas. 2017;103:45–53. 28778332 10.1016/j.maturitas.2017.06.025

[49] Yeasmin N, Akhter QS, Mahmuda S, et al. Association of hypertension with serum estrogen level in postmenopausal women. Mymensingh Med J. 2017;26(3):635–641. 28919621

[50] Martinelli S, Nannini G, Cianchi F, et al. The impact of microbiota–immunity–hormone interactions on autoimmune diseases and infection. Biomedicines. 2024;12(3):616. doi: 10.3390/biomedicines12030616 38540229 PMC10967803

[51] Arredondo A, Àlvarez G, Isabal S, et al. Comparative 16S rRNA gene sequencing study of subgingival microbiota of healthy subjects and patients with periodontitis from four different countries. J Clin Periodontol. 2023;50(9):1176–1187. doi: 10.1111/jcpe.13827 37246304

[52] Bertelsen RJ, Barrionuevo AMP, Shigdel R, et al. Association of oral bacteria with oral hygiene habits and self-reported gingival bleeding. J Clin Periodontol. 2022;49(8):768–781. doi: 10.1111/jcpe.13644 35569028 PMC9542802

