# Supplementary material for: Oral microbiome diversity and all-cause mortality in hypertensive adults: findings from a nationally representative cohort
Source: J Oral Microbiol. 2026 Jan 2;18(1):2609456. doi: 10.1080/20002297.2025.2609456 (PMC12777801; doi:10.1080/20002297.2025.2609456)
Supplement: Supplementary materials.docx [file ZJOM_A_2609456_SM8883.docx]

**Oral Microbiome Diversity and All-Cause Mortality in Hypertensive Adults: Findings from a Nationally Representative Cohort**

Zhe Zhou^1*^, Zichao Zhuang^1*^, Yipeng Ding^1^, Yufan Jiang^1^, Shu Chen^1^, Qinglian Zhang^1^, Hanxin Que^1^, Jian Lin^2^, Hui Deng^2^, Yi Wang^1,3^

Z. Zhou and Z. Zhuang contributed equally to this work.

1. Institute of Stomatology, School and Hospital of Stomatology, Wenzhou Medical University

2. Department of Periodontology, School and Hospital of Stomatology, Wenzhou Medical University

3. Department of Orthodontics, School and Hospital of Stomatology, Wenzhou Medical University

Correspondence:

Yi Wang, Institute of Stomatology, School and Hospital of Stomatology, Wenzhou Medical University, Email: [y.wang@wmu.edu.cn](mailto:y.wang@wmu.edu.cn)

Hui Deng, Department of Periodontology, School and Hospital of Stomatology, Wenzhou Medical University, Email: huideng@wmu.edu.cn

**Contents**

1. Fig. S1: Directed Acyclic Graph (DAG) for the Study of the Association Between Oral Microbiome and Mortality
2. Fig. S2: The pairwise Pearson correlation coefficient between different alpha diversity metrics
3. Fig. S3: Test for the proportional-hazards assumption using Schoenfeld residuals
4. Fig. S4. Forest plot of sex-stratified associations between oral microbiome diversity indices (Shannon–Weiner and Simpson) quartiles and all-cause mortality among hypertensive participants
5. Table S1: Mortality Distribution and Causes of Death During Follow-Up
6. Table S2: Adjusted Generalized Variance Inflation Factor (GVIF) Results for Multicollinearity Assessment of Variables Across Different Models with Oral Microbiome Diversity Indices
7. Table S3: Association Between β-Diversity Metrics and All-Cause Mortality Based on Principal Coordinates Analysis (PCoA1–PCoA4)
8. Table S4: Interaction Analysis of Sex, BMI, and Oral Microbiome Diversity (Simpson and Shannon-Weiner Indices) on All-Cause Mortality
9. Table S5: Sex-specific associations between oral microbiome diversity (per 1-SD increase) and mortality
10. Table S6: Sensitivity Analysis of the Association Between Oral Microbiome Diversity Indices and All-Cause Mortality After Excluding Participants with Cancer, Cardiovascular Diseases, and Metabolic-Related Conditions
11. Table S7: Association Between Oral Microbiome Diversity and All-Cause Mortality After Multiple Imputations and Multivariable Adjustment in Weighted Cox Regression

**Fig. S1: Directed Acyclic Graph (DAG) for the Study of the Association Between Oral Microbiome and Mortality.**

The DAG includes the exposure (oral microbiome diversity), potential confounders (age, sex, ethnicity, BMI, smoking status, drinking status, income, education, metabolic diseases, cardiovascular diseases, cancer, and periodontal disease), and the outcome (all-cause mortality). Arrows represent hypothesized causal pathways and were used to guide the selection of covariates for statistical adjustment


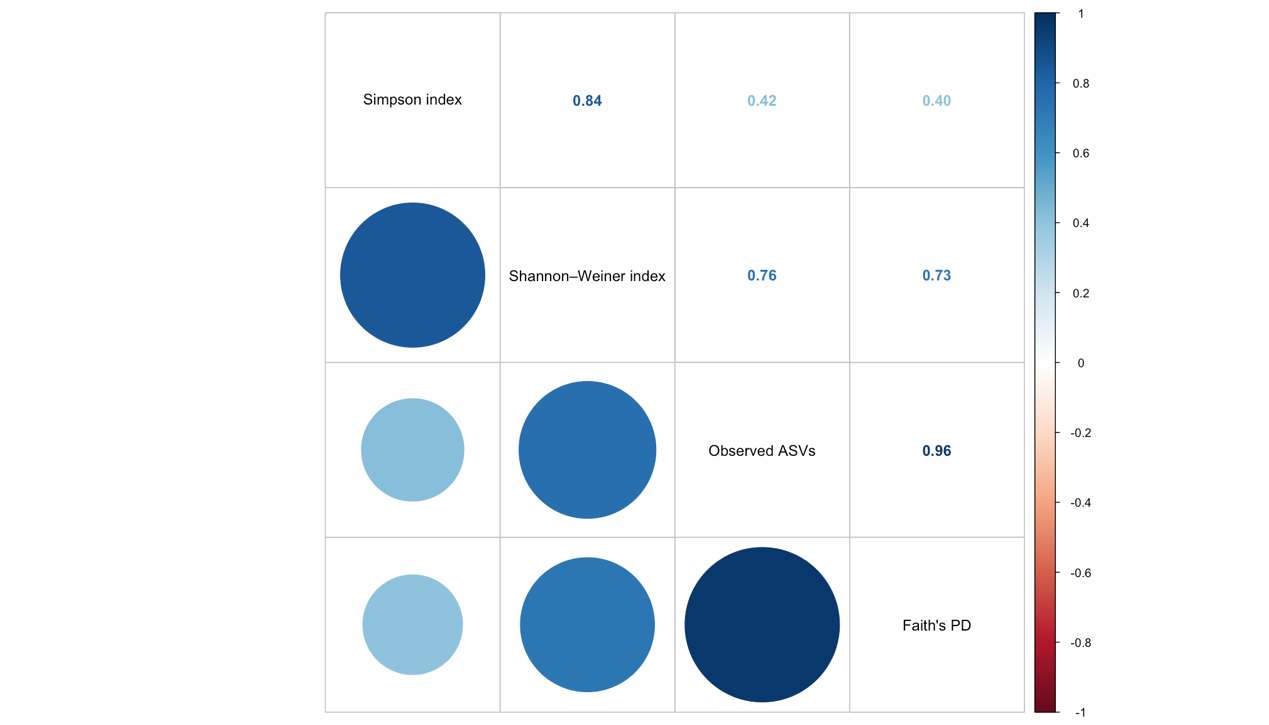


**Fig. S2: The pairwise Pearson correlation coefficient between different alpha diversity metrics.** Heatmap showing the degree of correlation between α-diversity indices

**_
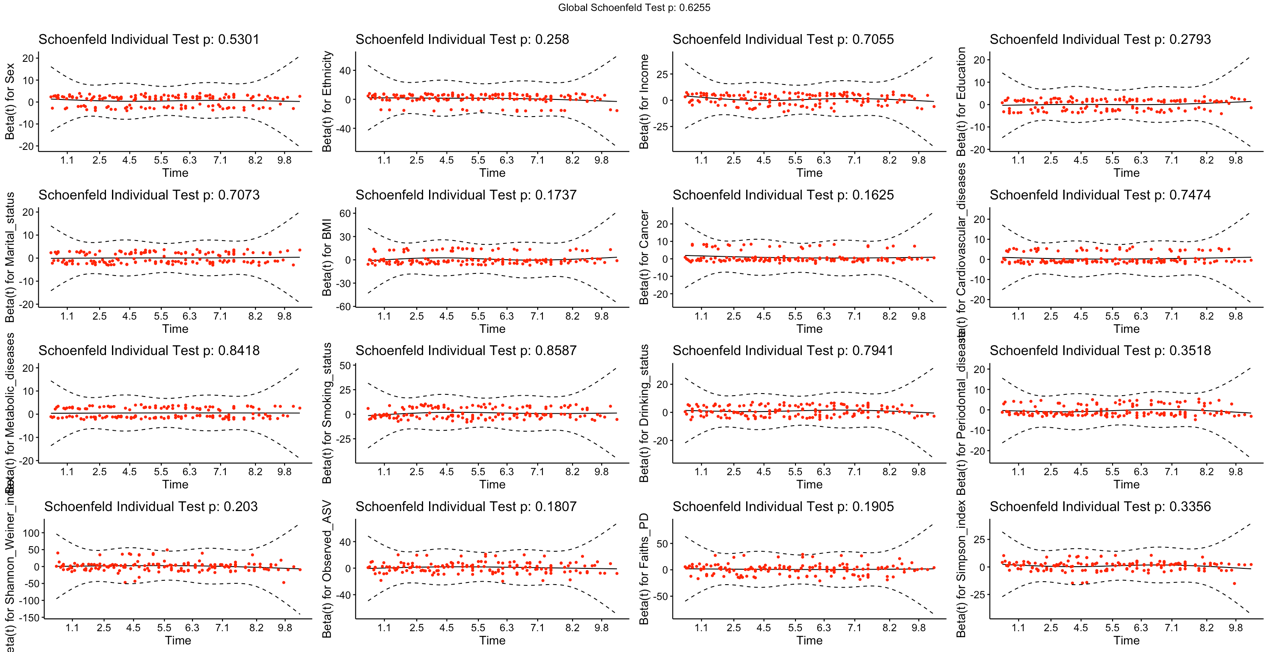
_**

**Fig. S3: Test for the proportional-hazards assumption using Schoenfeld residuals.** Proportional hazards assumption test for overall survival by plotting the Schoenfeld residuals against time. The X-axis represents the survival time (years). The Y-axis shows the Schoenfeld residuals referring to covariates, including Simpson index, Shannon–Weiner index, observed ASVs, Faith’s phylogenetic diversity, sex, ethnicity, income, education, marital status, smoking status, drinking status, BMI, periodontal disease, and comorbidities (cardiovascular diseases, metabolic-related diseases, and cancer). The constant mean of residuals across time confirms that the proportional hazards assumption holds for these covariates, with all *P*-values > 0.05. The global Schoenfeld test result is presented at the top of the plot, with *P* = 0.6255

**Fig. S4. Forest plot of sex-stratified associations between oral microbiome diversity indices (Shannon–Weiner and Simpson) quartiles and all-cause mortality among hypertensive participants.** Note: Models adjust for sex, ethnicity, income, education, marital status, smoking status, drinking status, BMI, periodontal disease, and comorbidities (cardiovascular diseases, metabolic-related diseases, and cancer), with age handled via left truncation

**Table S1: Mortality Distribution and Causes of Death During Follow-Up**

| **Cause of death** | **Number of people** |
| --- | --- |
| Diseases of heart | 70 |
| Malignant neoplasms | 76 |
| Chronic lower respiratory diseases | 11 |
| Accidents (unintentional injuries) | 8 |
| Cerebrovascular diseases | 13 |
| Alzheimer’s disease | 0 |
| Diabetes mellitus | 16 |
| Influenza and pneumonia | 8 |
| Nephritis, nephrotic syndrome and nephrosis | 2 |
| All other causes | 64 |
| Total | 268 |

Note: Follow-up and Mortality Data: During a total follow-up of 22,993 person-years (mean follow-up = 8.61 years)

**Table S2: Adjusted Generalized Variance Inflation Factor (GVIF) Results for Multicollinearity Assessment of Variables Across Different Models with Oral Microbiome Diversity Indices**

|  | **Adjusted Generalized Variance Inflation Factor** | | | |
| --- | --- | --- | --- | --- |
| Variable | Simpson index Model | Shannon–Weiner index Model | Faith's PD Model | Observed ASVs Model |
| Age | 1.14 | 1.15 | 1.16 | 1.16 |
| Gender | 1.10 | 1.10 | 1.11 | 1.10 |
| Ethnicity | 1.04 | 1.04 | 1.05 | 1.04 |
| Income Level | 1.09 | 1.09 | 1.09 | 1.09 |
| Education Level | 1.13 | 1.13 | 1.14 | 1.13 |
| Marriage | 1.07 | 1.07 | 1.07 | 1.07 |
| BMI | 1.03 | 1.03 | 1.03 | 1.03 |
| Cancer | 1.03 | 1.03 | 1.03 | 1.03 |
| Cardiovascular diseases | 1.04 | 1.04 | 1.04 | 1.04 |
| Metabolic syndrome | 1.05 | 1.05 | 1.05 | 1.05 |
| Smoking Status | 1.08 | 1.08 | 1.08 | 1.08 |
| Drinking status | 1.05 | 1.05 | 1.05 | 1.05 |
| Periodontal status | 1.15 | 1.16 | 1.18 | 1.18 |
| Microbial diversity index | 1.01 | 1.02 | 1.04 | 1.04 |

Note: Generalized Variance Inflation Factor (GVIF) was used to assess multicollinearity among variables in multivariable models. Adjusted GVIF values were calculated as GVIF^(1/(2×Df)) to account for degrees of freedom. Models were constructed with different oral microbiome diversity indices (Simpson, Shannon, Observed ASVs, and Faith's PD) as the exposure variables, adjusting for age (as left truncation time scale), sex, ethnicity, income, education, marital status, smoking status, drinking status, BMI, periodontal disease, and comorbidities (cardiovascular diseases, metabolic-related diseases, and cancer). All adjusted GVIF values were below the threshold of 5, indicating acceptable multicollinearity in all models (values < 5 indicate acceptable multicollinearity, values between 5-10 suggest moderate multicollinearity, and values ≥ 10 indicate severe multicollinearity)

**Table S3: Association Between β-Diversity Metrics and All-Cause Mortality Based on Principal Coordinates Analysis (PCoA1–PCoA4)**

| **Oral microbiome diversity** | **Model 1** | **Model 2** | **Model 3** |
| --- | --- | --- | --- |
|  | **HR (95% CI) per 1-SD** | **HR (95% CI) per 1-SD** | **HR (95% CI) per 1-SD** |
| **Bray-Curtis** |  |  |  |
| PCoA1 | 1.03 [0.86-1.23] | 0.95 [0.77-1.16] | 0.80 [0.62-1.03] |
| PCoA2 | 0.64 [0.54-0.75] | 0.70 [0.59-0.83] | 0.71 [0.58-0.88] |
| PCoA3 | 1.07 [0.90-1.28] | 0.98 [0.81-1.17] | 1.06 [0.82-1.36] |
| PCoA4 | 1.01 [0.82-1.23] | 1.02 [0.83-1.26] | 1.15 [0.87-1.52] |
| **Unweighted UniFrac** |  |  |  |
| PCoA1 | 1.14 [0.95-1.37] | 1.16 [0.97-1.39] | 1.10 [0.83-1.46] |
| PCoA2 | 1.34 [1.13-1.58] | 1.14 [0.94-1.38] | 0.97 [0.72-1.31] |
| PCoA3 | 0.86 [0.73-1.00] | 0.87 [0.74-1.01] | 0.95 [0.77-1.18] |
| PCoA4 | 0.97 [0.81-1.18] | 1.08 [0.88-1.31] | 1.02 [0.80-1.31] |
| **Weighted UniFrac** |  |  |  |
| PCoA1 | 0.70 [0.59-0.82] | 0.75 [0.63-0.89] | 0.76 [0.59-0.99] |
| PCoA2 | 0.88 [0.74-1.04] | 0.94 [0.78-1.12] | 0.96 [0.77-1.19] |
| PCoA3 | 0.75 [0.61-0.91] | 0.77 [0.62-0.94] | 0.78 [0.61-1.01] |
| PCoA4 | 1.06 [0.89-1.27] | 1.03 [0.85-1.25] | 0.97 [0.74-1.27] |

Note:

MV1: Adjusted for sex, with age accounted for by left truncation (using age as the time scale)

MV2: Additionally adjusted for ethnicity, income, education, marital status, smoking status, drinking status, and BMI, with age handled via left truncation

MV3: Additionally adjusted for periodontal disease and comorbidities (cardiovascular diseases, metabolic-related diseases, and cancer), with age accounted for by left truncation

**Table S4: Interaction Analysis of Sex, BMI, and Oral Microbiome Diversity (Simpson and Shannon-Weiner Indices) on All-Cause Mortality**

| **Variable** | **Interaction P for Sex** | **Interaction P for BMI** |
| --- | --- | --- |
| **Simpson index** | 0.77 | 0.34 |
| **Shannon–Weiner index** | **0.01** | 0.99 |

Note: Models adjust for sex, ethnicity, income, education, marital status, smoking status, drinking status, BMI, periodontal disease, and comorbidities (cardiovascular diseases, metabolic-related diseases, and cancer), with age handled via left truncation

**Table S5: Sex-specific associations between oral microbiome diversity (per 1-SD increase) and mortality**

| **Variable** | **HR (95% CI) per 1-SD — Female** | **HR (95% CI) per 1-SD — Male** |
| --- | --- | --- |
| **Simpson index** | 0.62 [0.51-0.76] | 0.91 [0.78-1.07] |
| **Shannon–Weiner index** | 0.53 [0.37-0.76] | 0.84 [0.66-1.07] |

Note: Models adjust for sex, ethnicity, income, education, marital status, smoking status, drinking status, BMI, periodontal disease, and comorbidities (cardiovascular diseases, metabolic-related diseases, and cancer), with age handled via left truncation

**Table S6:** **Sensitivity Analysis of the Association Between Oral Microbiome Diversity Indices and All-Cause Mortality After Excluding Participants with Cancer, Cardiovascular Diseases, and Metabolic-Related Conditions**

| **Oral microbiome diversity** | **Excluding Individuals with Cancer** | **Excluding Individuals with Metabolic-Related Diseases** | **Excluding Individuals with CVDs** |
| --- | --- | --- | --- |
|  | **HR (95% CI)** | **HR (95% CI)** | **HR (95% CI)** |
| Simpson index |  |  |  |
| Q1 | Ref. | Ref. | Ref. |
| Q2 | 0.78 [0.36-1.70] | 0.78 [0.33-1.86] | 0.49 [0.24-1.01] |
| Q3 | 0.47 [0.23-0.99] | 0.44 [0.18-1.06] | 0.32 [0.15-0.69] |
| Q4 | 0.43 [0.21-0.89] | 0.25 [0.10-0.62] | 0.32 [0.16-0.63] |
| p trend | < 0.01 | < 0.001 | < 0.01 |
| Shannon–Weiner index |  |  |  |
| Q1 | Ref. | Ref. | Ref. |
| Q2 | 0.57 [0.27-1.22] | 0.51 [0.21-1.23] | 0.40 [0.19-0.84] |
| Q3 | 0.59 [0.29-1.23] | 0.65 [0.28-1.48] | 0.55 [0.26-1.15] |
| Q4 | 0.48 [0.24-0.96] | 0.30 [0.14-0.64] | 0.41 [0.20-0.84] |
| *p* trend | 0.06 | < 0.05 | < 0.05 |
| Faith's PD |  |  |  |
| Q1 | Ref. | Ref. | Ref. |
| Q2 | 0.59 [0.27-1.31] | 0.60 [0.23-1.52] | 0.63 [0.28-1.44] |
| Q3 | 0.41 [0.20-0.85] | 0.67 [0.29-1.55] | 0.57 [0.26-1.27] |
| Q4 | 0.59 [0.28-1.21] | 0.53 [0.24-1.20] | 0.54 [0.23-1.28] |
| *p* trend | 0.10 | 0.19 | 0.16 |
| Observed ASVs |  |  |  |
| Q1 | Ref. | Ref. | Ref. |
| Q2 | 0.76 [0.35-1.65] | 0.71 [0.26-1.93] | 0.73 [0.33-1.64] |
| Q3 | 0.55 [0.25-1.19] | 0.84 [0.35-2.02] | 0.68 [0.30-1.52] |
| Q4 | 0.68 [0.34-1.35] | 0.59 [0.26-1.35] | 0.60 [0.27-1.32] |
| *p* trend | 0.19 | 0.33 | 0.21 |

Note: Models adjust for sex, ethnicity, income, education, marital status, smoking status, drinking status, BMI, periodontal disease, and comorbidities (cardiovascular diseases, metabolic-related diseases, and cancer), with age handled via left truncation

**Table S7: Association Between Oral Microbiome Diversity and All-Cause Mortality After Multiple Imputations and Multivariable Adjustment in Weighted Cox Regression**

| **Oral microbiome diversity** | **Model 1** | **Model 2** | **Model 3** |
| --- | --- | --- | --- |
|  | **HR (95% CI)** | **HR (95% CI)** | **HR (95% CI)** |
| Simpson index |  |  |  |
| Q1 | Ref. | Ref. | Ref. |
| Q2 | 0.76 [0.49-1.16] | 0.85 [0.53-1.36] | 0.90 [0.55-1.47] |
| Q3 | 0.49 [0.31-0.79] | 0.58 [0.35-0.98] | 0.63 [0.37-1.09] |
| Q4 | 0.36 [0.21-0.61] | 0.38 [0.21-0.68] | 0.41 [0.23-0.73] |
| *p* trend | <0.001 | <0.001 | <0.01 |
| Shannon–Weiner index |  |  |  |
| Q1 | Ref. | Ref. | Ref. |
| Q2 | 0.60 [0.38-0.95] | 0.61 [0.37-1.03] | 0.66 [0.40-1.11] |
| Q3 | 0.58 [0.36-0.92] | 0.65 [0.38-1.10] | 0.70 [0.40-1.20] |
| Q4 | 0.52 [0.33-0.83] | 0.50 [0.30-0.85] | 0.53 [0.31-0.92] |
| *p* trend | <0.01 | <0.05 | <0.05 |
| Faith's PD |  |  |  |
| Q1 | Ref. | Ref. | Ref. |
| Q2 | 0.66 [0.41-1.05] | 0.67 [0.39-1.14] | 0.72 [0.42-1.23] |
| Q3 | 0.62 [0.39-0.96] | 0.57 [0.35-0.95] | 0.59 [0.36-0.99] |
| Q4 | 0.74 [0.46-1.19] | 0.63 [0.38-1.05] | 0.69 [0.40-1.18] |
| *p* trend | 0.12 | <0.05 | 0.09 |
| Observed ASVs |  |  |  |
| Q1 | Ref. | Ref. | Ref. |
| Q2 | 0.68 [0.43-1.07] | 0.81 [0.47-1.38] | 0.88 [0.51-1.52] |
| Q3 | 0.56 [0.34-0.91] | 0.61 [0.35-1.04] | 0.65 [0.37-1.14] |
| Q4 | 0.74 [0.47-1.18] | 0.72 [0.43-1.19] | 0.77 [0.44-1.32] |
| *p* trend | 0.09 | 0.09 | 0.18 |

Note:

MV1: Adjusted for sex, with age accounted for by left truncation (using age as the time scale)

MV2: Additionally adjusted for ethnicity, income, education, marital status, smoking status, drinking status, and BMI, with age handled via left truncation

MV3: Additionally adjusted for periodontal disease and comorbidities (cardiovascular diseases, metabolic-related diseases, and cancer), with age accounted for by left truncation
